# Cytomegalovirus Retinitis Across the Immunosuppression Spectrum: A Contemporary Framework for Risk Stratification, Diagnosis, and Vision-Preserving Therapy

**DOI:** 10.3390/jcm15197482

**Published:** 2026-09-25

**Authors:** Shuai Su, Yulin Yang, Jiaying Wu, Yi Xiao

**Affiliations:** Department of Hematology, Tongji Hospital, Tongji Medical College, Huazhong University of Science and Technology, Wuhan 430030, China; 13569829324@163.com (S.S.); yyl05300@163.com (Y.Y.);

**Keywords:** cytomegalovirus, cytomegalovirus retinitis, HIV, immunosuppression, valganciclovir, ganciclovir, intravitreal therapy, antiviral resistance, immune recovery uveitis, retinal detachment

## Abstract

Cytomegalovirus retinitis (CMVR) causes irreversible visual loss in patients with severe cellular immunodeficiency. Among people living with human immunodeficiency virus (HIV), risk is the highest with profound CD4+ T-cell depletion and absent or ineffective antiretroviral therapy. Other susceptible groups include transplant recipients, patients receiving treatment for malignancy or targeted immunosuppression, and children with inborn errors of immunity. This narrative review connects risk assessment, diagnosis, antiviral selection, and follow-up. Dilated retinal examination and serial imaging establish lesion activity and vision threat. Ocular-fluid polymerase chain reaction supports uncertain diagnoses, but results require interpretation alongside retinal findings and prior treatment. Antiviral selection requires concurrent assessment of lesion location, diagnostic certainty, renal function, marrow reserve, and systemic cytomegalovirus (CMV) disease. Systemic valganciclovir or ganciclovir provides the treatment backbone when feasible; immediately sight-threatening lesions may require adjunctive intravitreal therapy. Resistance or toxicity can necessitate alternatives, while evidence for newer agents remains limited. Maintenance duration depends on retinal inactivity and sustained immune recovery, with less certain stopping criteria outside HIV. Surveillance should address recurrence, immune recovery uveitis, retinal detachment, macular and vascular injury, and visual dysfunction beyond central acuity. Geographically diverse studies illustrate detection gaps, although heterogeneous populations and methods limit direct comparisons of burden and treatment outcomes.

## 1. Introduction

Cytomegalovirus retinitis (CMVR) is a necrotizing retinal infection caused by human cytomegalovirus (CMV), a betaherpesvirus that persists in latent form and can reactivate when cell-mediated antiviral surveillance fails [1]. Its immediate threat is structural: viral replication causes full-thickness retinal necrosis and vascular injury, and lesions extending toward the fovea or optic disc can produce irreversible visual loss before systemic disease is fully recognized. The fundus phenotype therefore remains the diagnostic anchor, while molecular assays and multimodal imaging are used to resolve uncertainty rather than replace clinical examination.

The epidemiology has changed in both frequency and composition. Durable combination antiretroviral therapy (ART) has sharply reduced the incidence of acquired immunodeficiency syndrome (AIDS)-related CMVR, but risk persists in late presenters with human immunodeficiency virus (HIV) infection, patients with interrupted ART or incomplete virologic suppression, and people without timely access to retinal care. CMVR is also encountered in hematopoietic stem-cell and solid-organ transplant recipients, patients receiving cytotoxic or immune-targeted therapy, and those exposed to prolonged corticosteroids [2]. Clinical assessment should therefore characterize the host by the mechanism, depth, and expected duration of immune dysfunction rather than by HIV status alone.

ART has also altered the natural history after the initial episode. In the prospective five-year study of 503 patients with AIDS and CMVR, retinitis progression occurred at 7.0 per 100 person-years and retinal detachment at 2.3 per 100 eye-years [3]. These estimates are event rates, not cumulative percentages. Once viral replication is controlled, visual outcome depends on lesion topography, retinal detachment, macular injury, immune recovery uveitis (IRU), ischemia, and treatment toxicity [4]. Management must therefore extend from rapid retinal control to immune restoration and sustained ophthalmic follow-up.

Diagnosis begins with recognition of a compatible necrotizing retinitis phenotype in a susceptible host, followed by systematic exclusion of mimics. The Standardization of Uveitis Nomenclature effort has formalized classification criteria for CMVR, reinforcing the centrality of characteristic retinitis and the role of virologic confirmation in uncertain cases [5]. Yet classification criteria are not the same as a bedside algorithm: treatment urgency depends on whether lesions threaten the fovea or optic disc, whether the disease is progressing, and whether systemic antiviral exposure is safe. The evidence base spans landmark AIDS trials, ART-era cohorts, contemporary non-HIV series, and current practice guidance.

## 2. Scope and Literature Search

This clinically oriented narrative review addresses acquired CMVR across HIV-associated and non-HIV immunodeficiency. Shuai Su conducted a targeted literature search in PubMed/MEDLINE on 19 September 2026, using the controlled term Cytomegalovirus Retinitis and the free-text terms cytomegalovirus retinitis and CMV retinitis. Searches covered publications until September 2026. Topic-specific searches combined these terms with polymerase chain reaction (PCR), children, pediatric, immunodeficiency, transplantation, visual field, electroretinography, retinal detachment, macular edema, and geographic terms. Separate searches addressed immune-function assessment and clinical electrophysiology standards. PubMed Central and publisher websites were used for full-text retrieval; cited reference lists and exact-title or DOI searches supplemented identification. Supplementary File S1 gives the executed search strings and retrieval details.

English-language human clinical reports relevant to screening, diagnosis, antiviral treatment, immune assessment, or ocular outcomes were considered. Randomized trials and prospective cohorts were prioritized for treatment effects and natural history. Retrospective studies and case reports were retained for uncommon pediatric, immunodeficiency, and salvage-treatment settings. Guidelines, consensus statements, and systematic reviews provided context and recommendations; primary reports were used for study-specific numerical estimates. Reports limited to anterior-segment CMV disease, congenital infection without relevant active retinitis, or nonclinical drug development were excluded from the clinical synthesis. This targeted update did not constitute exhaustive systematic screening, duplicate independent selection, or a new meta-analysis. Selection and publication bias therefore remain possible. Search transparency, source attribution, and distinction of evidence levels follow the reporting principles addressed by Scale for the Assessment of Narrative Review Articles (SANRA) [6].

The review is arranged around the clinical decisions shown in Figure 1. Section 3, Section 4 and Section 5 and Table 1 identify susceptible hosts and screening needs; Section 6 and Table 2 address retinal diagnosis, ocular PCR, imaging, and visual function. Section 7 and Table 3 support antiviral selection and monitoring, while Table 4 summarizes the supporting studies without ranking regimens across different populations. Section 8 addresses nonresponse and resistance. Section 9 covers immune recovery, complications, and maintenance withdrawal; Section 10 connects these components at the first visit and subsequent reviews. Figure 2 places selected cohorts geographically and preserves their different denominators. The pathway integrates published guidance with clinical interpretation and has not been prospectively validated as a decision rule [7].

**Figure 1:**
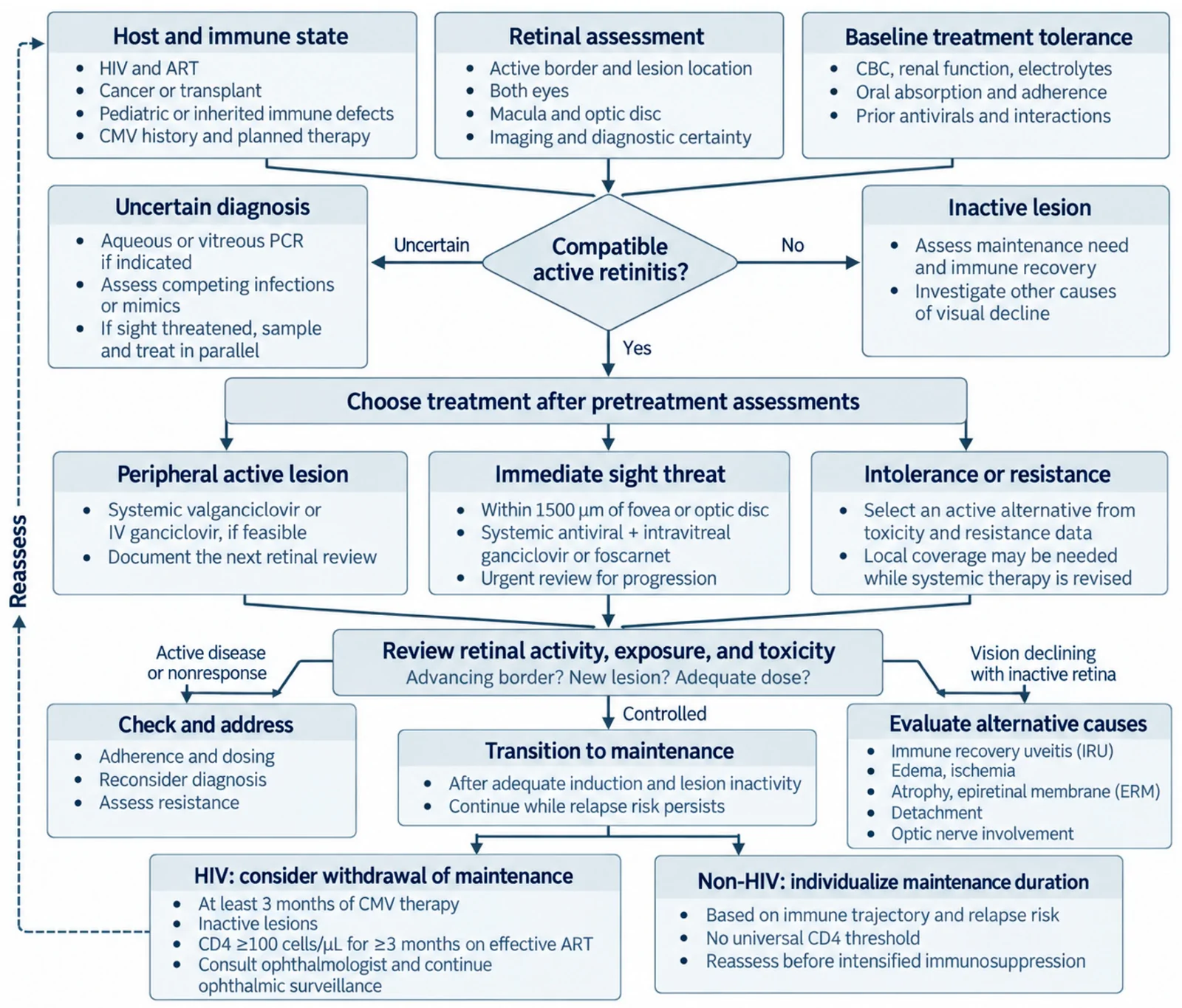
Clinical pathway for suspected or established cytomegalovirus retinitis. Host immune status, retinal activity and location, diagnostic certainty, and baseline treatment tolerance are assessed before antiviral selection. For a clinically typical sight-threatening lesion, diagnostic sampling and treatment can proceed in parallel. Systemic therapy provides fellow-eye and extraocular coverage; intravitreal ganciclovir or foscarnet supplements it when immediate retinal exposure is required. Inactive disease leads to maintenance and host-specific reassessment, whereas progression or visual decline triggers evaluation for inadequate exposure, resistance, recurrent infection, or structural and inflammatory complications. Solid arrows indicate clinical sequence; the labeled return arrow indicates reassessment. Section and table references direct readers to Supplementary Materials. This author-constructed synthesis is informed by HIV and transplant guidance and CMVR studies, rather than a validated prediction model [3,7,8,9]. ART, antiretroviral therapy; CBC, complete blood count; CMV, cytomegalovirus; ERM, epiretinal membrane; HIV, human immunodeficiency virus; IRU, immune recovery uveitis; IV, intravenous; PCR, polymerase chain reaction.

**Figure 2:**
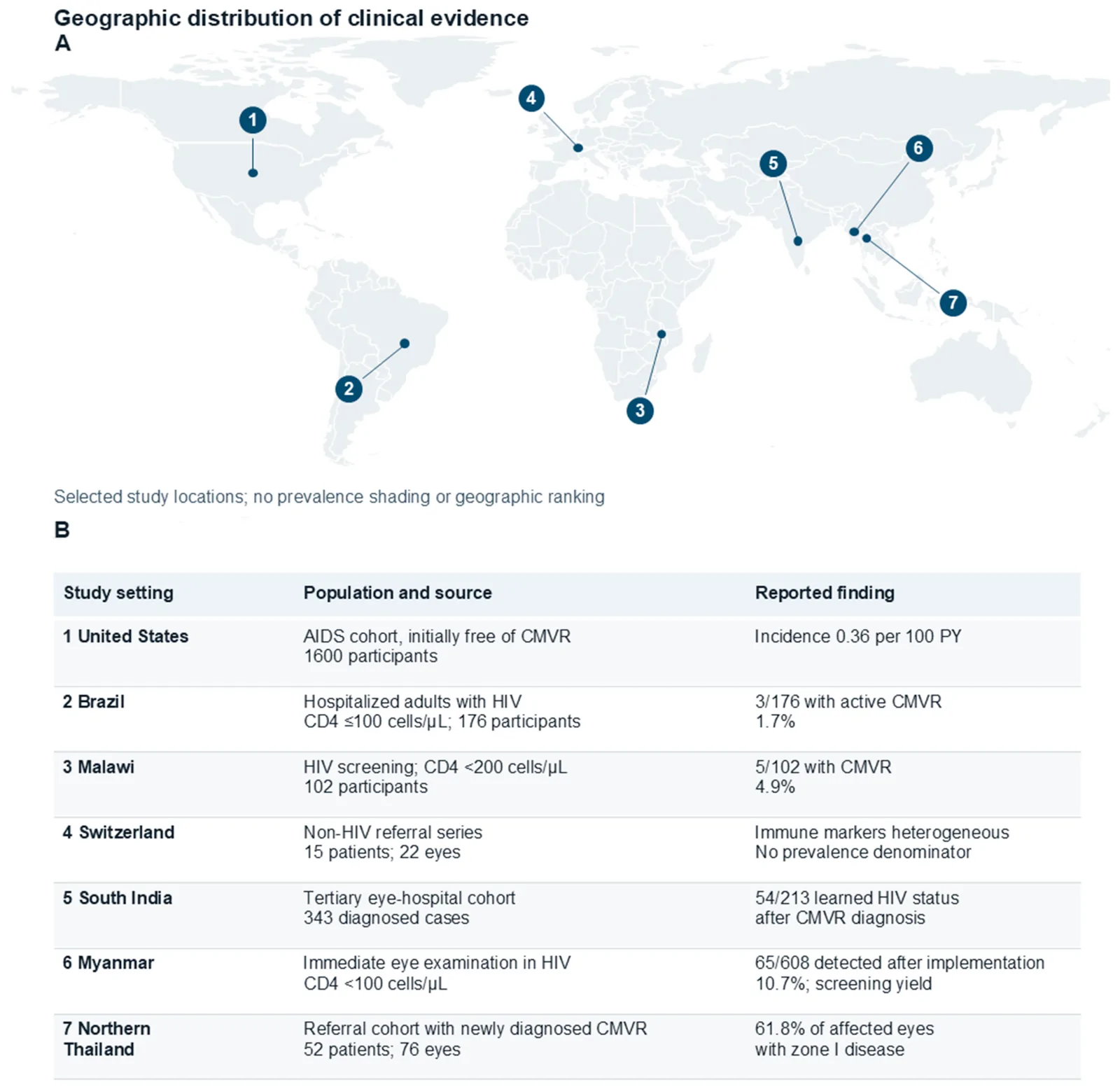
Geographic distribution of selected clinical evidence on cytomegalovirus retinitis. (**A**), Study settings from the Americas, Europe, Africa, and Asia. (**B**), The corresponding populations and reported findings. The map shows the locations of selected studies, not country-level prevalence or a ranking of current hotspots. The United States value is an incidence rate; the Brazilian, Malawian, and Myanmar values are proportions detected in selected HIV populations; the Swiss, Indian, and Thai studies are case series or referral cohorts. Differences in CD4 eligibility, ART exposure, screening intensity, calendar period, and referral selection prevent direct comparison. Country markers indicate approximate locations, not exact catchment boundaries. Data sources are Sugar et al. [10] (United States), Ausayakhun et al. [11] (Thailand), Passarin et al. [12] (Switzerland), Ocieczek et al. [13] (Malawi), Ei et al. [14] (Myanmar), Rathinam et al. [15] (South India), and Bogoni et al. [16] (Brazil). The base map uses public-domain Natural Earth boundaries. No new clinical estimates or pooled analysis were generated. ART, antiretroviral therapy; CMVR, cytomegalovirus retinitis; HIV, human immunodeficiency virus; PY, person-years.

**Table 1:** Host-risk-stratified prevention and screening framework for cytomegalovirus retinitis.

Host Context	Risk Signals	Preventive/Screening Response	Key Limitation
Advanced HIV	CD4 <100 cells/µL for baseline assessment; CD4 <50 cells/µL, ineffective ART or new visual symptoms increase concern	Baseline ophthalmic examination below CD4 100; repeat every 3–4 months below 50; urgent examination for new symptoms; optimize ART	Guideline-supported HIV thresholds; peripheral disease can be asymptomatic [7]
Allogeneic HSCT	Persistent/recurrent CMV DNAemia, graft-versus-host disease, corticosteroid escalation, lymphopenia, prior CMV disease	Apply transplant CMV prevention; lower threshold for ophthalmic examination during prolonged CMV replication or new visual symptoms	Blood CMV clearance does not prove absence of established retinal disease [17,18,19,20]
Solid-organ transplantation	High-risk serostatus, breakthrough/recurrent CMV, intense immunosuppression, diabetes or other coexisting risks	Continue transplant-specific prophylaxis/pre-emptive monitoring; targeted retinal examination when ocular risk is elevated	Universal eye screening has uncertain yield [21,22]
Hematologic malignancy/targeted therapy	Chemotherapy, prolonged corticosteroids, B-cell depletion, JAK inhibition, prior CMV disease, severe lymphopenia	Medication-specific vigilance; visual-symptom counseling; rapid retinal examination; individualized screening when multiple risk factors coexist	No validated single CD4 or CMV viral-load threshold across heterogeneous non-HIV hosts [23,24,25,26,27,28,29,30,31]
Children with IEI or malignancy/HSCT	Age-adjusted lymphopenia, combined immune defects, CMV DNAemia, continuing immunosuppression; symptoms may be absent	Pediatric ophthalmology and immunology input; risk-directed repeat assessment; anticipate limits of symptom reporting and examination	No universal pediatric screening interval or adult CD4 stopping threshold [32,33,34,35,36,37]
Previously treated CMVR	Prior extensive lesions, ongoing immunosuppression, unstable immune recovery, prior recurrence	Secondary prophylaxis when indicated; bilateral follow-up; detect retinal breaks, detachment, IRU and macular complications; withdrawal criteria in Section 9.4	Risk persists after virologic quiescence [3,38,39,40,41]

Abbreviations: ART, antiretroviral therapy; CD4, cluster of differentiation 4; CMV, cytomegalovirus; CMVR, cytomegalovirus retinitis; DNA, deoxyribonucleic acid; HIV, human immunodeficiency virus; HSCT, hematopoietic stem-cell transplantation; IRU, immune recovery uveitis; JAK, Janus kinase; RNA, ribonucleic acid; SOT, solid-organ transplantation. IEI, inborn errors of immunity.

**Table 2:** Diagnostic hierarchy for suspected cytomegalovirus retinitis.

Diagnostic Domain	Findings That Support CMVR	How It Changes Management	Pitfalls
Clinical retina examination	Granular or confluent necrotizing retinitis; hemorrhage; perivascular spread; active whitening at lesion border; compatible immunosuppression	Treat a characteristic active, sight-threatening lesion promptly; assess baseline toxicity in parallel, then select treatment with Table 3	Atypical non-HIV inflammation and ischemic variants can mimic other retinitides [5,42,43]
Serial color/wide-field imaging	Border stabilization or regression during treatment; new satellite lesion or expanding border during relapse	Provides anatomical definition of response and failure	Interpret serial borders and new lesions; a stable scar is not active CMVR [44]
OCT/angiographic imaging	Full-thickness retinal disruption, macular edema, epiretinal membrane, capillary nonperfusion or occlusive vasculopathy	Identifies structural cause of vision loss and complications such as IRU or ischemia	Findings characterize complications but are not pathogen-specific [44,45]
Aqueous or vitreous PCR	CMV DNA with compatible retinitis; test competing pathogens when indicated	Resolve atypical disease or treatment failure; consider repeat/different sampling after an unexplained negative test	Negative results do not exclude CMVR; low-level positivity alone does not prove activity; no universal stopping threshold [46,47,48]
Blood CMV DNA/antigenemia	Supports systemic CMV replication and increases pretest probability in some hosts	Useful for systemic risk assessment and resistance genotyping if sufficiently positive	Negative blood testing does not exclude CMVR; blood clearance is not a retinal endpoint [49,50,51,52,53]

Abbreviations: CMV, cytomegalovirus; CMVR, cytomegalovirus retinitis; DNA, deoxyribonucleic acid; HIV, human immunodeficiency virus; IRU, immune recovery uveitis; OCT, optical coherence tomography; PCR, polymerase chain reaction.

**Table 3:** Antiviral treatment options and their clinical roles in cytomegalovirus retinitis.

Agent and Route	Usual Role	Adult Regimen or Practical Use	Main Constraints
Valganciclovir, oral	Systemic induction and maintenance when absorption is reliable	900 mg twice daily for 14–21 days, then 900 mg daily after retinal control [7,9]	Myelosuppression; renal adjustment; adherence and absorption
Ganciclovir, intravenous	Induction when oral delivery is unreliable or disease is severe	5 mg/kg every 12 h for 14–21 days; transition after response [7,9]	Myelosuppression; renal adjustment; venous access
Ganciclovir, intravitreal	Adjunct for immediate sight threat	2 mg per injection, usually weekly during induction; individualized by retinal response [7,54]	No fellow-eye/systemic protection; injection and compounding risks
Foscarnet, intravenous	Ganciclovir resistance or treatment-limiting cytopenia	60 mg/kg every 8 h or 90 mg/kg every 12 h for induction [7,55,56]	Renal toxicity; calcium, magnesium, potassium and phosphate disturbances; hydration
Foscarnet, intravitreal	Local adjunct or salvage while systemic therapy is revised	2.4 mg per injection, usually weekly during induction [7]	Injection risks; no fellow-eye/systemic protection
Cidofovir, intravenous	Selected intolerance or resistance when other options are unsuitable	Specialist-directed therapy with saline and probenecid [7,57,58]	Nephrotoxicity, uveitis and hypotony; intravitreal use is contraindicated
Letermovir, oral or intravenous	Established transplant prophylaxis; limited off-label ocular experience	Do not substitute prophylactic efficacy for evidence in active retinitis [8,59,60]	Resistance and interaction concerns; uncertain ocular exposure
Maribavir, oral	Exceptional salvage reports; consensus advises avoidance in active CMVR	Small CMVR case series only; systemic trial excluded retinitis [61,62]	Uncertain ocular efficacy; interactions and resistance; avoid ganciclovir/valganciclovir combination [8]

Regimen examples refer to adults with HIV-associated CMVR and normal renal function. They require renal adjustment where applicable and are not pediatric dosing instructions. Induction continues for at least 14–21 days and until the retinal response is adequate; a calendar duration alone is insufficient. Non-HIV and pediatric regimens require specialist individualization. Maintenance continuation and withdrawal are discussed in Section 7.5 and Section 9.4. CMVR, cytomegalovirus retinitis; HSCT, hematopoietic stem-cell transplantation; IV, intravenous.

**Table 4:** Selected quantitative clinical evidence informing contemporary CMVR management.

Study/Evidence Source	Population	Key Quantitative Result	Clinical Interpretation
LSOCA prospective cohort [10]	1600 AIDS participants without CMVR	29 incident cases/8134 PY; 0.36/100 PY overall; 3.89/100 PY when CD4 <50 cells/µL; HR 136	Risk enrichment is concentrated in profound cellular immunodeficiency.
Five-year ART-era cohort [3]	503 patients with AIDS and CMVR	Progression 7.0/100 PY; retinal detachment 2.3/100 EY; visual loss to ≤20/200 3.4/100 EY	Retinal complications remain clinically important after ART-era treatment.
Valganciclovir RCT [9]	160 patients with newly diagnosed AIDS-related CMVR	Four-week progression 7/71 with oral valganciclovir vs. 7/70 with IV ganciclovir; all switched to oral maintenance after week 4	Comparable induction efficacy in AIDS; no placebo maintenance comparison.
Intravitreal ganciclovir meta-analysis, 2026 [54]	18 studies; 1132 eyes	Anatomic resolution 89%; first-week exposure ≥ 4000 µg vs. lower exposure: 94% vs. 73%, subgroup *p* = 0.019	Study-level association from mainly case series; no significant dose-group difference in vision, recurrence or detachment.
Diverse immunocompromised cohort, 2026 [63]	91 patients; 117 eyes	Grade +2 vitritis 34.8% non-HIV vs. 19.7% HIV-positive; baseline VA 1.31 vs. 1.85 logMAR; RD independently predicted worse final VA	Phenotypic differences; baseline acuity and detachment associated with final vision in this cohort. No validated prognostic score.
Maribavir case series, 2026 [62]	6 patients; 11 eyes	4/11 eyes active at switch; those 4 became quiescent within 6 weeks; 7 already inactive remained inactive	Uncontrolled salvage experience; cannot establish comparative efficacy or first-line benefit.

Abbreviations: AIDS, acquired immunodeficiency syndrome; ART, antiretroviral therapy; CD4, cluster of differentiation 4; CMVR, cytomegalovirus retinitis; EY, eye-years; HIV, human immunodeficiency virus; HR, hazard ratio; IV, intravenous; logMAR, logarithm of the minimum angle of resolution; LSOCA, Longitudinal Study of the Ocular Complications of AIDS; PY, person-years; RD, retinal detachment; RCT, randomized controlled trial; VA, visual acuity.

## 3. Epidemiology and the Changing Host Spectrum

### 3.1. HIV-Associated CMVR in the ART Era

Prospective data show that risk remains sharply concentrated in advanced cellular immunodeficiency. In the Longitudinal Study of the Ocular Complications of AIDS (LSOCA), 1600 participants without CMVR were followed for 8134 person-years; 29 incident cases occurred, corresponding to 0.36 cases per 100 person-years overall [10]. The rate rose to 3.89 per 100 person-years after visits at which the CD4+ T-cell count was below 50 cells/µL, and this threshold was associated with a hazard ratio of 136 (95% CI, 30–605) for subsequent retinitis. Only three incident cases occurred when the preceding CD4+ count was ≥50 cells/µL [10]. CD4+ count is therefore most useful for risk enrichment rather than for defining retinal activity.

CMVR was historically described as a neglected component of the AIDS pandemic because patients often presented only after visual symptoms had developed and ophthalmic services were poorly integrated with HIV programs [64]. Experience from China and Southeast Asia reached a similar conclusion: inexpensive fundus examination can identify a treatable cause of blindness only when referral pathways and trained examiners are available [65]. Effective prevention therefore requires an operational bridge between HIV care and eye care, not simply access to antiviral drugs.

Long-term ART-era follow-up shows that ocular risk persists despite improved systemic control. Among 503 patients with AIDS and CMVR, overall mortality was 9.8 per 100 person-years, retinitis progression 7.0 per 100 person-years, retinal detachment 2.3 per 100 eye-years, and visual loss to 20/200 or worse 3.4 per 100 eye-years [3]. Patients with newly diagnosed CMVR had the highest rates of progression (28.0 per 100 person-years) and retinal detachment (4.9 per 100 eye-years). The baseline retinal examination should therefore document not only whether CMVR is present but also the extent and location of retinal injury.

Vision loss is not determined solely by whether CMV replication is suppressed. Lesion proximity to the macula or optic nerve, baseline visual acuity, retinal detachment, and inflammatory complications all shape the final outcome [66]. Retinal detachment is especially consequential in settings where vitreoretinal surgery is unavailable or delayed; resource-limited cohorts identify extensive retinitis and other markers of retinal destruction as major risk factors [67]. Prevention must therefore include prevention of late presentation and prevention of structural complications after virologic control.

The conceptual model established during the ART era remains useful: treatment has a local objective, to halt retinal border advancement, and a systemic objective, to reduce contralateral-eye and extraocular CMV risk while immune recovery is being achieved [68]. Failure to control viral replication can have systemic implications. Resistant CMV in patients with AIDS and retinitis has been associated with increased mortality, reflecting both the biological severity of prolonged CMV replication and the limited salvage options available in earlier eras [69].

Five-year observational data demonstrate that recurrence, retinal detachment, and visual impairment remain possible long after the first episode, particularly when immune recovery is incomplete or unstable [3]. Second-eye involvement and retinal detachment are therefore not historical curiosities; they are clinically relevant competing risks that justify systemic therapy when feasible and continued bilateral examination even when only one eye is symptomatic [38].

Untreated AIDS-related CMVR typically progresses at the active border, with patterns ranging from hemorrhagic necrosis along retinal vessels to more granular peripheral disease [70]. ART-era Brazilian experience likewise showed that clinical presentation is heterogeneous and that outcomes depend on both systemic HIV control and ocular extent at diagnosis [71]. This heterogeneity is why symptom-only strategies are insufficient: peripheral CMVR can be asymptomatic until substantial retina has been destroyed.

ART-era studies include patients with different treatment exposures and should not be read as cohorts of uniformly suppressed HIV. Tang and colleagues evaluated hospitalized people with HIV during 2010–2016 and described ocular and immunologic changes after ART [72]. In the northern Thai cohort, 90.4% had started ART, but lesion size suggested that retinitis preceded ART in every patient [11]. These findings describe advanced disease around entry to treatment, rather than substantial residual CMVR risk during durable virologic suppression. CD4 count and blood CMV DNA inform host risk, but neither establishes retinal activity [49,73].

The distinction between a systemic risk marker and an ocular diagnostic endpoint is clinically decisive. CMV viremia has been associated with disease progression and mortality in HIV, and viral DNA may fall after effective ART even without CMV-specific therapy [50,51]. Blood CMV DNA can therefore raise suspicion and guide systemic management, but a declining or negative blood assay does not establish retinal quiescence; the retinal border remains the relevant endpoint for vision.

### 3.2. CMVR Beyond HIV

HIV-negative CMVR occurs across heterogeneous immune defects. In a Swiss series of 15 patients with non-HIV CMVR, only 4 of 12 patients with available measurements had CD4 counts below 100 cells/µL; hypogammaglobulinemia was present in 7 of 11 tested patients [12]. These small denominators preclude a validated screening threshold. Earlier comparative studies also described greater ocular inflammation and different clinical trajectories outside HIV [74]. Risk assessment should incorporate the underlying disease, cumulative immunosuppression, and expected immune recovery, including functional testing when the clinical history suggests a broader immune defect.

Direct comparisons of HIV-positive and HIV-negative CMVR demonstrate overlapping retinal necrosis but differences in vitreous inflammation, host age, comorbidity, and treatment context [75]. Visual prognosis may be worse in some non-HIV cohorts because of delayed recognition, greater inflammatory response, systemic malignancy, or limited ability to reduce immunosuppression [76]. More recent prognostic analyses support a multifactorial model in which lesion location, baseline vision, immune status, and complications interact [77].

A 2026 retrospective cohort of 91 patients (117 eyes) further illustrates biological differences between HIV-positive and HIV-negative CMVR. Non-HIV eyes were more likely to have grade +2 vitritis than HIV-positive eyes (34.8% vs. 19.7%, *p* = 0.009), whereas HIV-positive eyes presented with worse mean baseline visual acuity (1.85 ± 1.14 vs. 1.31 ± 1.17 logMAR, *p* = 0.045) [63]. Among non-HIV patients, retinal vascular sheathing was more frequent in the drug-induced immunosuppression subgroup (30% vs. 0%, *p* = 0.009). Baseline visual acuity and retinal detachment independently predicted final visual acuity, while the underlying immunosuppression category was not significantly associated with final vision in this cohort. Reports of CMV-associated posterior uveitis and panuveitis in non-HIV patients further support this broader inflammatory phenotype [78].

At the severe end of this spectrum, chronic retinal necrosis has been described as a CMV-associated necrotizing retinitis with prominent panretinal vasculopathy in non-HIV immunosuppressed patients [79]. Retinal arteriolar occlusion can also dominate the presentation in older HIV-negative adults [80]. These vascular phenotypes matter because a clinician who expects only the classic “pizza-pie” hemorrhagic lesion may misclassify ischemic or minimally hemorrhagic disease.

Long-term follow-up of non-HIV CMVR shows that recurrence remains possible while immunosuppression persists and that the endpoint for maintenance therapy is less clearly defined than in ART-responsive HIV [39]. Lymphoma-associated CMVR illustrates this complexity: chemotherapy, malignancy-related immune dysfunction, marrow reserve, and the need for continuing oncologic treatment all influence antiviral choice and duration [81]. In these hosts, prevention depends on repeated reassessment of risk as immunosuppression changes rather than on a universal CD4 threshold.

### 3.3. Transplantation, Hematologic Malignancy, and Targeted Immunosuppression

After allogeneic hematopoietic stem-cell transplantation (HSCT), systemic CMV reactivation is sufficiently common that structured surveillance and pre-emptive therapy are embedded in routine transplant practice. CMVR is uncommon relative to CMV viremia but is associated with recognizable risk factors, including prolonged or recurrent CMV replication and profound immune dysfunction [17]. Active ophthalmic screening triggered by CMV antigenemia has identified retinitis before later symptomatic presentation [18], although the optimal screening threshold and interval remain uncertain.

Solid-organ transplantation (SOT) and hematopoietic stem-cell transplantation differ in the timing and pattern of immunosuppression, but both can be complicated by CMVR in the setting of systemic CMV replication [82]. In a 2026 case–control study of solid-organ transplant recipients, diabetes mellitus was associated with CMVR, but the adjusted estimate was imprecise: odds ratio 16.5, with a 95% confidence interval of 1.12–243.8 [21]. This association does not justify universal retinal screening after transplantation, but it supports a lower threshold for examination when metabolic disease coexists with breakthrough or recurrent CMV replication and intensive immunosuppression.

Earlier transplant series showed that CMVR may present late in the post-transplant course and threaten vision despite systemic monitoring [22]. More recent allogeneic HSCT studies have refined ocular prognostic factors [19] and risk factors for CMVR occurrence [20], again implicating persistent CMV replication, sustained immunosuppression, and delayed immune reconstitution. A negative systemic assay should therefore be interpreted in temporal context rather than treated as evidence that established retinal disease is absent.

Prolonged glucocorticoid exposure increases the risk of a broad range of opportunistic infections, including herpesvirus reactivation, through dose- and duration-dependent suppression of cellular immunity [23]. B-cell depletion with rituximab produces a different immunologic defect but is also associated with serious infections, especially when combined with corticosteroids, cytotoxic agents, or baseline hypogammaglobulinemia [24]. Persistent hypogammaglobulinemia and late-onset neutropenia can further complicate infection risk after rituximab [25]. These mechanisms support medication-history review as a core element of CMVR risk stratification.

Janus kinase (JAK) inhibition merits specific attention because CMVR has been reported with more than one agent. Tofacitinib-associated disease has been described in rheumatoid arthritis [26], and CMVR followed by IRU has also been reported after methotrexate plus tofacitinib [27]. In 2026, CMVR was reported during upadacitinib monotherapy [28], and another JAK-inhibitor case involved retinal ischemia and vitreous hemorrhage [29]. These reports remain too sparse to support class-wide screening recommendations, but they justify direct counseling about floaters, blurred vision, scotoma, and photopsias and a low threshold for dilated examination when additional immunosuppressive risks are present.

### 3.4. Children and Inborn Errors of Immunity

Children may have advanced bilateral disease before they can describe visual symptoms. In a historical series of nine immunosuppressed children, only two reported visual symptoms at presentation and eight had bilateral CMVR; the mixed cohort included severe combined immunodeficiency, leukemia, transplantation, HIV, and congenital infection [32]. Acquired CMVR should therefore be considered in children with profound or persistent cellular immune dysfunction, including during apparently stable phases of treatment for malignancy. Congenital CMV-associated chorioretinal disease is a distinct clinical setting and is not treated here as epidemiologically interchangeable with acquired opportunistic CMVR.

Inborn errors of immunity associated with reported CMVR include severe combined immunodeficiency, combined T- and B-cell defects, and Wiskott–Aldrich syndrome [32,33,34]. In the ten-patient Wiskott–Aldrich series, CMVR occurred before and after HSCT, with some children represented in both periods [33]. Suspected disease warrants coordinated pediatric ophthalmology, immunology, and infectious-disease assessment. Age-adjusted lymphocyte reference ranges, immune phenotype, CMV history, and the timing of transplantation or immune replacement matter more than application of an adult HIV CD4 threshold. Young age, developmental ability, and systemic illness may limit examination or require anesthesia for selected procedures; these practical constraints should be anticipated rather than allowed to postpone evaluation.

Among 228 pediatric allogeneic HSCT recipients in a single-center retrospective study, three developed CMVR; all were asymptomatic, and retinitis appeared 5–12 weeks after detection of DNAemia [35]. The study questioned the yield of universal early photography and supports risk-directed repeated assessment rather than a universal schedule. Outside transplantation, one CMVR case occurred among 50 children with acute lymphoblastic leukemia undergoing virologic monitoring, and high-level DNAemia was observed during maintenance chemotherapy [36]. These observations complement reports of CMVR during leukemia maintenance and of CD4 lymphopenia in affected children [30,31]. They do not establish a common screening cutoff. Antiviral dosing and toxicity monitoring require pediatric specialist input, with attention to body size, renal function, marrow suppression, and the feasibility of repeated intravitreal treatment.

### 3.5. Geographic Distribution of the Evidence

Published data indicate persistent clinical burden in parts of South and Southeast Asia and sub-Saharan Africa, but available studies do not define a contemporary worldwide hotspot ranking. In Malawi, screening identified CMVR in 5 of 102 people with HIV and CD4 counts below 200 cells/µL; all five affected patients had counts below 50 cells/µL [13]. In Myanmar, introduction of immediate eye examination for patients with CD4 counts below 100 cells/µL increased detected CMVR from 14 of 1233 before implementation to 65 of 608 afterward [14]. The difference demonstrates the influence of ascertainment and should not be interpreted as a temporal increase in underlying disease incidence. A separate Myanmar treatment cohort demonstrates delivery of oral and intravitreal therapy through primary HIV services [83].

Referral cohorts describe disease severity rather than population prevalence. In northern Thailand, 52 newly diagnosed patients contributed 76 affected eyes, of which 61.8% had zone I disease [11]. In South India, 343 patients were diagnosed during 2007–2023, including 322 with HIV; among 213 with a documented HIV diagnosis date, 54 learned their HIV status only after CMVR was diagnosed [15]. Earlier Indian cohorts likewise reported diverse and advanced presentations [84]. These data identify settings where delayed detection can cause preventable visual loss, but referral selection prevents direct regional comparison.

The geographic picture also includes lower measured burdens in other selected populations. A Brazilian hospital cohort found active CMVR in 3 of 176 severely immunosuppressed adults with HIV [16], while the United States LSOCA study reported 0.36 incident cases per 100 person-years among 1600 participants initially free of CMVR [10]. Non-HIV series, including the Swiss cohort, document disease during treatment for malignancy and transplantation without providing a population prevalence estimate [12]. The sub-Saharan African systematic review by Ismail and colleagues pooled ten studies with 1931 participants and estimated prevalence at 3.2%, with a 95% confidence interval of 1.6–5.4% [85]. This regional synthesis complements the primary cohorts; its variation in ART exposure and recruitment limits comparison with Asian referral series. Figure 2 locates selected primary studies and states each denominator and endpoint.

## 4. Host and Lesion Assessment

### 4.1. Pathobiology and Baseline Risk

CMV latency and reactivation reflect the balance between viral persistence and cell-mediated immune surveillance. Latently infected hematopoietic-lineage cells may contribute to viral dissemination when immune control is disrupted [86]. In the retina, direct cytopathic injury occurs alongside endothelial dysfunction, vascular occlusion, inflammatory necrosis, and breakdown of the blood–retinal barrier. The visible active border therefore functions as an integrated marker of ongoing viral replication and tissue injury; the therapeutic objective is to stop border advancement before the fovea or optic nerve is damaged.

The retinal phenotype also depends on the immunologic environment. Comparative clinical studies show that different immunosuppressive states produce different balances between viral replication and ocular inflammation [87]. Profound CD4+ depletion in AIDS may be accompanied by relatively little vitritis, whereas non-HIV patients may show more conspicuous vitritis, vasculitis, or anterior inflammation. A clinically useful risk model therefore incorporates four domains: the depth and mechanism of immune dysfunction, systemic CMV activity, retinal lesion location and extent, and the patient’s renal and marrow reserve for antiviral therapy.

CMV can also involve other ocular compartments, including the anterior chamber and corneal endothelium, and these syndromes should not be conflated simply because they share a viral cause [88]. CMVR is a posterior-segment necrotizing infection, and treatment decisions must be anchored to retinal activity. Conversely, CMV DNA detected in ocular fluid must be interpreted in the context of the phenotype because PCR positivity without compatible retinitis may reflect another CMV ocular syndrome or, rarely, low-level bystander DNA. Figure 1 places host, retinal, diagnostic, and baseline toxicity assessment together before treatment selection; these domains are then reassessed throughout therapy.

### 4.2. Functional Immune Assessment

Absolute cell counts incompletely describe immune competence in primary or complex secondary immunodeficiency. Selected patients warrant T- and B-cell phenotyping in addition to complete blood count (CBC), CD4 and CD8 counts, and serum immunoglobulins. Evaluation may include CD3, CD4, CD8, and CD19 populations; naive and memory T-cell distributions; and class-switched memory B cells, interpreted against age-adjusted reference ranges and recent corticosteroid, cytotoxic, or B-cell-depleting exposure [37]. The purpose is to characterize the host defect and guide immunologic care; no such panel has been validated as a standalone predictor of CMVR relapse.

Vaccine-specific antibody responses, such as historical or clinically appropriate responses to tetanus and pneumococcal antigens, assess humoral function. Their interpretation requires vaccination history and consideration of immunoglobulin replacement or other passive antibody exposure. They do not measure CMV-specific cellular control. Lymphocyte proliferation to mitogens and recall antigens assesses broad T-cell proliferative capacity and antigen responses, respectively; prior sensitization, lymphopenia, immunosuppressive treatment, specimen handling, and laboratory reference ranges affect interpretation [37]. Testing should be selected with an immunologist and should not delay antiviral treatment of active retinitis.

CMV-specific assays, including interferon-γ release, enzyme-linked immunospot (ELISpot) assay, or cytokine flow cytometry, address antiviral cellular responses more directly. Recurrent retinitis despite recovery of total CD4 counts has been associated with deficient CMV-specific CD4 responses in a small HIV case series [89]. Transplant trials have investigated immune-guided discontinuation of primary antiviral prophylaxis, but their systemic infection endpoints cannot establish a safe stopping threshold after CMVR; one randomized trial shortened prophylaxis but did not establish noninferiority for CMV infection [90]. Current use should therefore remain contextual. Decisions about CMVR maintenance withdrawal require retinal inactivity and the full immune trajectory, as discussed in Section 9.4 [8].

## 5. Prevention and Screening Strategies

### 5.1. Primary Prevention in HIV

Durable immune restoration with suppressive ART is the principal preventive intervention in HIV. Routine primary valganciclovir prophylaxis and routine blood CMV monitoring are not recommended solely to prevent CMV end-organ disease, including in patients with DNAemia; the randomized AIDS Clinical Trials Group (ACTG) A5030 study did not establish a benefit sufficient to support that approach [7,91]. Prevention should combine HIV diagnosis, effective ART, symptom education, and timely retinal examination.

Current National Institutes of Health (NIH) guidance recommends a baseline ophthalmologic examination for people with HIV and CD4 counts below 100 cells/µL. When CD4 counts remain or fall below 50 cells/µL, examinations every 3–4 months are appropriate because peripheral disease may be asymptomatic [7]. New floaters or visual change require urgent assessment regardless of the scheduled interval. Implementation must account for local capacity, but limited access should be identified as a service gap rather than treated as evidence that screening has no value.

### 5.2. Prevention in Transplantation and Other Immunosuppressed States

In HSCT and SOT, CMV prevention is already structured around donor–recipient serostatus, antiviral prophylaxis or pre-emptive monitoring, quantitative nucleic-acid testing, and adjustment of immunosuppression. Ophthalmic surveillance should be layered onto this strategy selectively. Persistent or recurrent CMV DNAemia, CMV end-organ disease, corticosteroid escalation, graft-versus-host disease, profound lymphopenia, or visual symptoms should lower the threshold for retinal examination. Because CMVR may become apparent after systemic markers improve, a preceding period of high CMV replication should remain part of the risk history even after blood PCR becomes negative. This is a risk-directed clinical approach; validated universal ophthalmic screening thresholds remain unavailable [8,17,18,52].

Standardized ocular screening protocols are less developed for hematologic malignancy and targeted immune therapies. A pragmatic approach is to recognize coexisting risk factors: active CMV replication with major T-cell dysfunction, multiple immunosuppressive agents, prolonged high-dose corticosteroids, prior CMV disease, or new visual symptoms. In such patients, the procedural burden of a dilated retinal examination is modest relative to the consequence of missing central retinitis. Medication reconciliation should specifically capture recent JAK inhibition, B-cell depletion, T-cell-directed therapy, transplantation, and chemotherapy because patients may no longer describe themselves as immunocompromised after leaving the inpatient setting. Pediatric risk assessment is addressed separately in Section 3.4 [35,36].

### 5.3. Preventing Ocular Damage After CMVR Is Diagnosed

Secondary prevention begins at the first retinal examination. The immediate goals are to keep active necrosis away from zone 1, protect the fellow eye and extraocular sites with systemic therapy when feasible, limit antiviral toxicity through renal and hematologic monitoring, and prevent late structural complications such as retinal detachment, macular edema, epiretinal membrane, and ischemia. Long-term cohort data show that retinal detachment and second-eye involvement remain clinically important even after antiviral therapy [3,38].

When a susceptible patient has a characteristic lesion threatening the macula or optic nerve, ocular sampling and antiviral initiation can proceed in parallel [7]. A positive or negative blood PCR should not delay treatment of clinically established retinitis. Intravitreal prophylaxis in an eye without retinitis has no established role; the role of local therapy in active disease is described in Section 7.2.

Table 1 organizes host-specific screening and follow-up, and Figure 1 connects those decisions to retinal confirmation and treatment. Screening identifies patients who need examination; treatment selection additionally requires lesion activity, vision threat, and baseline renal and hematologic assessment.

## 6. Diagnosis and Visual Assessment

### 6.1. Clinical Morphology and Anatomic Staging

Diagnosis remains primarily clinical. Classic CMVR may appear as granular or confluent retinal whitening, hemorrhagic necrosis tracking along vessels, and relatively little vitreous inflammation in advanced AIDS [42]. The active border is more informative than the central atrophic scar; serial wide-field photography can determine whether the lesion has stopped advancing and distinguish a stable scar from a new edge of activity.

A 2026 tertiary eye-center series further demonstrates the heterogeneity of presentation and the value of structured description [43]. Each eye should be characterized by lesion location and extent, distance from the fovea and optic disc, vitreous inflammation, hemorrhage, vascular occlusion, retinal breaks, and detachment. Immediate sight threat should be distinguished from broader anatomic zone I classification: lesions within 1500 µm of the fovea or optic disc meet the NIH treatment definition for adjunctive intravitreal therapy [7].

The principal infectious differential diagnoses are acute retinal necrosis caused by varicella-zoster virus or herpes simplex virus and ocular toxoplasmosis. Comparative studies of herpetic retinitis identify differences in lesion distribution, inflammation, vascular involvement, and host context, but substantial overlap remains [92]. Syphilitic retinitis, progressive outer retinal necrosis, fungal chorioretinitis, intraocular lymphoma, and noninfectious occlusive vasculitis may also mimic CMVR. Atypical features should prompt targeted testing rather than diagnostic delay.

### 6.2. Ocular Fluid PCR and the Limits of Blood Testing

Ocular-fluid PCR is particularly useful when the fundus appearance is atypical, the media obscure the lesion, a competing infection is plausible, or presumed CMVR progresses despite adequate treatment [46]. Aqueous sampling is often the initial approach; vitreous sampling may be appropriate when suspicion remains high after a negative aqueous result or when vitrectomy is otherwise indicated. Testing for CMV should be combined with herpes simplex virus (HSV), varicella-zoster virus (VZV), and Toxoplasma gondii when these organisms are plausible. Syphilis testing and investigation for lymphoma or other noninfectious mimics remain separate diagnostic tasks. Marked inflammation in an HIV-negative host increases the need for careful differential diagnosis, but HIV-negative status alone does not mandate an invasive test.

PCR performance depends on the population, specimen, assay, and treatment timing. In one AIDS cohort, CMV DNA was detected in aqueous fluid in 46 of 49 patients with active CMVR, compared with 7 of 49 blood samples [47]. These figures should not be generalized to all hosts or laboratories. Low viral burden, a small sample, PCR inhibition, and prior antiviral exposure can produce a negative result; persistent clinical suspicion may justify repeat sampling or a different ocular specimen [46]. Conversely, detection of viral DNA can persist in clinically inactive disease, so positivity alone does not establish ongoing necrosis [48].

No universally validated aqueous or vitreous viral-load cutoff separates active retinitis from inactive infection or defines treatment completion. Quantitative trends are most interpretable when specimen type and laboratory method are consistent. Changes should be assessed alongside lesion borders, new retinal lesions, imaging, and treatment exposure. Low-level positivity with an incompatible phenotype should prompt review for another ocular CMV syndrome, an alternative pathogen, or a noninfectious diagnosis; a positive CMV assay does not exclude coinfection. Serial invasive sampling is reserved for cases in which the result can change management [5,46,47,48,88].

Systemic CMV DNAemia is useful for risk stratification in transplantation and hematology, but it cannot substitute for retinal examination. A 2025 study of screening examinations among patients with CMV viremia highlights the practical question: which viremic patients have a sufficiently high pretest probability to justify ophthalmic screening [52]? The answer is likely context-specific and depends on immune phenotype, viral kinetics, symptoms, and local CMVR prevalence rather than on a universal viral-load threshold.

Conversely, CMVR may become clinically apparent after CMV antigenemia has converted to negative during systemic antiviral therapy [53]. The eye can behave as a partially compartmentalized site, and systemic exposure sufficient to suppress blood replication may still have been inadequate in duration, concentration, or timing to prevent established retinal disease. Recent work examining routes of systemic infection in HIV-associated CMVR further supports a model in which blood markers inform but do not define ocular infection [93].

### 6.3. Multimodal Imaging

Color fundus photography, preferably ultra-widefield when available, documents lesion borders and peripheral involvement. The 2026 Multimodal Imaging in Uveitis consensus identifies clinical examination and color photography as the principal tools for diagnosis and follow-up [44]. Optical coherence tomography (OCT) characterizes retinal layer injury, macular edema, epiretinal membrane, and atrophy; fundus autofluorescence can help delineate lesion borders. Fluorescein angiography is useful for vascular occlusion, macular leakage, and optic-disc leakage. OCT angiography can demonstrate microvascular abnormalities, but its role in assessing CMVR activity remains limited [44,45].

Imaging should be tied to a defined management question. At baseline, wide-field photography establishes the lesion map and OCT documents macular involvement, edema, or structural loss. During induction, imaging determines whether the active border has stopped advancing; during maintenance, it helps distinguish recurrent whitening from post-necrotic change; after immune recovery, OCT can identify IRU-associated macular edema even when retinitis is inactive. Used this way, imaging becomes a longitudinal outcome measure rather than an additional diagnostic layer collected without a decision attached. These modalities characterize anatomy and complications; they do not identify a pathogen independently [44].

Table 2 links the presenting retinal findings to the next diagnostic step. A compatible active lesion can justify treatment, whereas atypical retinitis requires targeted microbiologic or alternative diagnostic assessment. Baseline CBC, renal function, medication review, and immune assessment then inform the treatment options in Table 3 and Figure 1.

### 6.4. Visual Function Beyond Central Acuity

Central visual acuity can remain useful while substantial peripheral retina has been lost. Static or kinetic perimetry documents scotoma extent and can complement serial photography when the patient can perform reliable testing [94]. Long-term LSOCA follow-up also measured visual-field loss and showed that functional outcomes differed with immune recovery [95]. Automated central fields may suit paracentral defects, whereas kinetic testing can capture wider peripheral loss. Test selection should reflect lesion distribution, age, fatigue, and fixation; a new field defect requires renewed retinal examination.

Electrophysiology is a selective adjunct when reported vision, acuity, and structural findings are discordant or when reliable behavioral testing is difficult. Full-field electroretinography (ERG) measures generalized retinal function, while multifocal ERG maps localized cone-driven retinal responses, particularly in the posterior pole [96,97]. Visual evoked potential (VEP) evaluates transmission through the visual pathway and can assist assessment of suspected optic-nerve or postretinal dysfunction [98]. Macular injury, media opacity, poor fixation, and age can affect these recordings and complicate localization. International Society for Clinical Electrophysiology of Vision (ISCEV) standards define test acquisition and interpretation; they do not establish CMVR-specific thresholds for antiviral escalation or withdrawal. These tests may document residual damage and inform rehabilitation after infection control, without delaying urgent treatment.

## 7. Antiviral Treatment and Monitoring

### 7.1. Treatment Goals and Evidence Base

Treatment selection requires simultaneous assessment of retinal threat, diagnostic certainty, renal and marrow reserve, oral absorption, adherence, and prior antiviral exposure. The systematic review by Putera and colleagues synthesizes a broad treatment literature but also documents substantial heterogeneity in host population, regimen, era, and outcome definition [99]. Randomized CMVR trials answer narrower questions and should retain their original comparators and endpoints. Systemic CMV trials are identified separately because blood viral clearance cannot establish retinal efficacy.

The pivotal valganciclovir trial randomized 160 patients with newly diagnosed AIDS-related CMVR to oral valganciclovir or intravenous ganciclovir for induction [9]. Among evaluable participants, progression within four weeks occurred in 7 of 71 receiving valganciclovir and 7 of 70 receiving intravenous ganciclovir, corresponding to 9.9% and 10.0%. All participants received oral valganciclovir for maintenance after week 4. This trial therefore supports comparable induction efficacy. The maintenance phase did not compare different randomized regimens. Its small number of early progression events and AIDS population limit inference about subgroup superiority or non-HIV outcomes.

Before valganciclovir became available, oral ganciclovir maintenance showed that systemic suppressive therapy could delay progression after induction [100]. Combining a sustained-release intraocular ganciclovir implant with oral ganciclovir also demonstrated the enduring local-systemic principle: high intraocular exposure controls the treated eye, whereas systemic therapy reduces fellow-eye and extraocular disease [101]. Although the implant is no longer central to current practice, the same logic supports combining intravitreal injection with systemic therapy when rapid local control is needed.

The historical foscarnet-versus-ganciclovir trial remains informative because it demonstrated comparable control of retinitis while exposing important differences in systemic outcomes and toxicity. Among 234 patients, 65 assigned to ganciclovir died compared with 36 assigned to foscarnet (51% vs. 34%; relative risk 1.79, 95% CI 1.17–2.73), with a median survival of 8.5 versus 12.6 months [102]. Retinitis progression did not differ, but the survival difference should be interpreted within the premodern ART treatment context, including unequal ART exposure, rather than attributed solely to antiviral toxicity. In the ART era, foscarnet is best regarded as a toxicity- or resistance-directed alternative rather than a routine first-line substitute for ganciclovir.

### 7.2. Local Therapy for Sight-Threatening Disease

Adjunctive intravitreal treatment is considered when lesions immediately threaten central vision or progress rapidly. Historical implant trials established the value of sustained high ocular drug exposure, but the ganciclovir implant is no longer manufactured and is not included in the contemporary treatment pathway [7,103,104]. Local therapy supplements systemic treatment when feasible because an intravitreal injection does not protect the fellow eye or extraocular organs.

For immediately sight-threatening lesions, NIH guidance recommends intravitreal ganciclovir 2 mg per injection or foscarnet 2.4 mg per injection in addition to systemic treatment, repeated weekly during induction until inactivity [7]. The regimen may be individualized for progression, toxicity, or resistance by the treating retinal specialist. Intravitreal cidofovir is contraindicated because of severe ocular toxicity. Standardized preparation and dose verification are essential; the concentration, injected volume, and total weekly exposure must be documented separately.

Cidofovir remains effective but is limited by nephrotoxicity and ocular adverse effects. In the HPMPC randomized trial, 64 patients with previously untreated small peripheral CMVR lesions were randomized to deferred therapy or low- or high-dose cidofovir [57]; subsequent comparative work evaluated cidofovir against an implant-plus-oral-ganciclovir strategy [58]. Its historical efficacy explains why cidofovir retains a salvage role, but the toxicity profile and monitoring requirements make it less attractive than valganciclovir or foscarnet for routine induction. When it is used, probenecid, hydration, renal monitoring, and surveillance for uveitis and hypotony are essential.

Jeong and colleagues specifically examined intravitreal ganciclovir dosing in a 2026 meta-analysis of 18 studies comprising 1132 eyes [54]. Pooled anatomic resolution was 89%, and studies with cumulative first-week exposure of at least 4000 µg had higher resolution than those with lower exposure, 94% versus 73%, with a subgroup *p* value of 0.019. There was no significant dose-group difference in visual outcome, recurrence, or retinal detachment. The comparison was largely based on heterogeneous case series, with differences in injection frequency, host immunity, concurrent systemic therapy, and follow-up. It therefore supports further evaluation of early exposure, not a causal claim that increasing a single injection dose improves vision. Lower- and higher-dose cohorts should be read in their original clinical contexts [105,106].

Experience in HIV-negative patients with lymphoma also shows that combined oral and intravitreal antiviral therapy can control CMVR while lymphoma treatment continues [107]. The clinical challenge is that retinal quiescence may be followed by renewed risk when chemotherapy or corticosteroids are intensified again. Antiviral planning should therefore anticipate the future immunosuppressive trajectory rather than respond only to the current retinal appearance.

### 7.3. Systemic Therapy, Monitoring, and Toxicity Management

Ganciclovir and valganciclovir inhibit viral DNA polymerase after phosphorylation by the CMV UL97 kinase. Their main clinical liabilities are neutropenia, anemia, thrombocytopenia, renal dose dependence, and potential reproductive toxicity. Before induction, clinicians should document renal function, complete blood count, concurrent myelosuppressive drugs, and the feasibility of serial monitoring. A falling neutrophil count is clinically consequential because it may force dose interruption, increase bacterial or fungal infection risk, and create conditions for inadequate CMV exposure and resistance. Coordination with hematology or transplant teams is therefore part of antiviral management rather than an administrative add-on. Established safety guidance informs these assessments [7,8].

Foscarnet bypasses UL97 activation and can retain activity against many UL97-mediated ganciclovir-resistant strains. Its principal liabilities are nephrotoxicity, electrolyte wasting, genital ulceration, infusion burden, and the need for careful hydration and laboratory monitoring. Systemic foscarnet is particularly useful when marrow toxicity precludes ganciclovir or when genotypic resistance predicts ganciclovir failure, but prolonged outpatient use may be difficult. Intravitreal foscarnet can provide immediate ocular coverage while a systemic strategy is revised, provided that fellow-eye and extraocular risks are also addressed. Route-specific advantages and limitations are summarized in Table 3 [7,55,56].

During induction, retinal activity and systemic tolerance should be reviewed together. Compare photographs for advancing borders or new lesions, record acuity and symptoms, and verify dosing, adherence, oral absorption, CBC, renal function, and relevant electrolytes. HIV guidance specifies examination at diagnosis, at approximately two weeks, and monthly thereafter; more frequent review is appropriate for sight-threatening progression and is inherent in weekly intravitreal treatment [7]. Non-HIV schedules should be individualized to the lesion and immune trajectory. Persistent activity despite adequate exposure prompts the structured evaluation in Section 8.

### 7.4. Letermovir, Transplant Prophylaxis, and the Boundary Between Prevention and Treatment

Letermovir inhibits the CMV terminase complex and lacks the marrow toxicity of ganciclovir, making it attractive in patients with hematologic disease. Small ocular series have described its use in CMV-associated uveitis and retinitis [59], but evidence for active CMVR remains far less mature than that for ganciclovir, valganciclovir, or foscarnet. The relatively low genetic barrier to resistance in some treatment settings and uncertain ocular pharmacokinetics argue against letermovir monotherapy for sight-threatening active CMVR when established alternatives are feasible.

In HSCT, by contrast, letermovir has a strong prevention evidence base. A randomized trial demonstrated effective prophylaxis against clinically significant CMV infection after hematopoietic-cell transplantation [60]. Reducing systemic CMV reactivation can indirectly reduce ocular risk, but prophylactic success should not create false reassurance: breakthrough CMV and rare CMVR still occur, and visual symptoms still require retinal examination. Prevention of viremia and diagnosis of retinitis are related but distinct clinical tasks.

For SOT, current international consensus guidance emphasizes risk-stratified CMV prevention, quantitative viral-load monitoring, prophylaxis or pre-emptive strategies, and management of resistant or refractory infection [8]. Ophthalmologists should understand the transplant team’s CMV strategy because prior prophylaxis changes both the pretest probability of resistance and the interpretation of breakthrough viremia. Conversely, transplant clinicians should regard confirmed CMVR as end-organ disease with an ophthalmology-defined retinal endpoint rather than relying on blood viral clearance alone.

Route of administration is a major determinant of retinal exposure, systemic coverage, toxicity, and feasibility. Table 3 distinguishes oral, intravenous, and intravitreal delivery and summarizes the clinical role of each option. Selection follows the pretreatment assessment in Figure 1; Table 4 provides study-level evidence and does not function as a separate prescribing algorithm.

### 7.5. Practical Monitoring During Induction and Maintenance

Table 4 keeps the selected evidence alongside the monitoring and maintenance decisions that it informs. Each row specifies a source population and endpoint; estimates from different rows should not be compared as if they arose from a single randomized trial.

Antiviral monitoring should be protocolized because some apparent treatment failures are pharmacologic rather than virologic. For valganciclovir or intravenous ganciclovir, baseline and serial complete blood counts and renal function are essential, with shorter intervals when marrow reserve is poor, renal function is changing, or other myelotoxic drugs are being administered. Dose adjustment should follow renal function rather than an arbitrary response to cytopenia because underexposure may permit continued viral replication. When clinically feasible, neutropenia can be managed by modifying other marrow-suppressive agents or using hematopoietic growth-factor support in collaboration with the systemic care team. The objective is to preserve effective anti-CMV exposure while controlling toxicity, not to maintain a nominal drug regimen at a subtherapeutic dose [7,8].

Systemic foscarnet requires a different safety infrastructure. Serum creatinine, calcium, magnesium, potassium, and phosphate should be monitored closely, hydration optimized, and concomitant nephrotoxins minimized. Electrolyte abnormalities can develop rapidly and become clinically important before symptoms appear. Cidofovir requires even more restrictive use because nephrotoxicity is cumulative and ocular hypotony or uveitis may complicate treatment [57,58,102]. These toxicity profiles also explain the role of intravitreal therapy: in selected patients, it provides immediate retinal antiviral exposure while systemic treatment is being modified or rescued.

Intravitreal therapy requires monitoring beyond retinitis activity. Each injection visit should include a brief assessment for pain, redness, decreased vision, pressure change, vitreous haze, retinal tear, or detachment. Endophthalmitis is uncommon but vision-threatening, and repeated injections increase cumulative procedural exposure. For bilateral injections, separate preparation and strict aseptic technique help reduce the risk of bilateral catastrophic complications. A written compounding standard should specify drug concentration, withdrawal volume, final injected volume, labeling, storage, and independent verification; this systems approach is particularly important for high-dose ganciclovir protocols in which a decimal or concentration error can be consequential [54,105,106].

Maintenance begins after an adequate induction course and control of active retinitis; it suppresses recurrence while immune risk persists and is not automatically lifelong [7]. In HIV, withdrawal can be considered after at least three months of anti-CMV treatment, inactive retinal lesions, and CD4 recovery to at least 100 cells/µL for at least three months on ART, with ophthalmology agreement. Section 9.4 gives post-withdrawal monitoring and restart criteria. Non-HIV patients have no comparable validated immune threshold, so duration depends on ongoing immunosuppression and recurrence history [39,107]. Any new lesion or major increase in immunosuppression requires reassessment.

Multidisciplinary communication is integral to treatment. Ophthalmology should specify whether retinitis is active, which eye is threatened, whether intravitreal therapy is being administered, and what retinal finding will define control. Infectious-disease or transplant teams should communicate systemic viral kinetics, antiviral susceptibility, renal and marrow constraints, and the planned prophylactic strategy. Hematology, oncology, and rheumatology teams should clarify whether immunosuppression can be reduced and when chemotherapy, corticosteroids, or targeted agents will be intensified again. Explicit information sharing helps anticipate relapse windows and prevents contradictory decisions, such as stopping systemic antiviral therapy immediately before renewed intensive immunosuppression [17,18,21,26,27,28,29,81,82].

## 8. Refractory Disease and Antiviral Resistance

Refractory CMVR should be defined clinically before it is defined molecularly [108]. Apparent progression may reflect an inadequate induction interval, underdosing after incorrect renal adjustment, poor adherence, malabsorption, missed intravitreal treatment, immune-mediated inflammation, or an alternative retinal diagnosis. True antiviral resistance becomes more likely after prolonged viral replication under drug pressure, recurrent disease during suppressive therapy, extensive prior ganciclovir exposure, or lesion progression despite verified therapeutic dosing. CMV can accumulate mutations in viral kinase and polymerase pathways that alter drug susceptibility.

The molecular basis of resistance is clinically actionable. UL97 mutations classically reduce phosphorylation and activation of ganciclovir while preserving susceptibility to foscarnet and cidofovir, whereas UL54 DNA-polymerase mutations can produce broader cross-resistance patterns [55]. High-level ganciclovir resistance may involve alterations in both UL97 and UL54 [109], while foscarnet resistance maps to polymerase mutations in distinct domains and may emerge after sustained exposure [56]. Resistance genotypes should therefore be interpreted drug by drug rather than labeled generically as resistant CMV.

Historical CMVR cohorts showed that ganciclovir resistance becomes more frequent with increasing cumulative exposure [110]. In current practice, resistance testing is usually performed on blood CMV DNA when viremia is sufficient for genotyping; ocular-fluid genotyping may be considered for compartmentalized disease when blood testing is negative and a validated assay is available. Turnaround time is clinically important. When a lesion is advancing toward the fovea, empiric intravitreal foscarnet or another active local agent may be required before genotypic results return.

Maribavir, an oral UL97 kinase inhibitor with a mechanism distinct from ganciclovir activation, has expanded systemic options for refractory or resistant CMV after transplantation. In the phase 3 SOLSTICE trial, maribavir achieved superior CMV clearance compared with investigator-assigned therapy for refractory or resistant post-transplant infection [61]. Patients with CMV retinitis were excluded from that trial; its viral-clearance endpoint therefore does not establish treatment efficacy for active retinal infection.

The 2025 international transplant consensus advises avoiding maribavir for CMV retinitis because ocular efficacy is uncertain [8]. A subsequent six-patient case series reported control of four active eyes and continued quiescence in seven previously inactive eyes after transition to maribavir for resistance, toxicity, or intolerance [62]. This uncontrolled experience provides a salvage signal but does not overturn the guidance or establish first-line efficacy. Any exceptional use requires specialist justification, close retinal monitoring, and a clear plan for local control. Drug interactions, emergent resistance, and dysgeusia also require attention. Concomitant ganciclovir or valganciclovir should be avoided because maribavir inhibits the UL97-dependent activation of ganciclovir [8].

Adoptive CMV-specific cytotoxic T-lymphocyte therapy represents a different strategy: it seeks to restore pathogen-specific immunity. Ocular outcomes after adoptive immunotherapy have been reported in highly immunocompromised patients with CMVR [111]. These approaches remain specialized, but they underscore a broader principle: durable CMVR control ultimately depends on immune competence. Antivirals suppress replication; they do not eradicate latent CMV. Pediatric experience includes successful donor-derived CMV-specific T-cell treatment after HSCT, but evidence remains limited to specialized reports [112].

## 9. Immune Recovery, Recurrence, and Structural Complications

### 9.1. Immune Recovery Uveitis

IRU is intraocular inflammation associated with immune recovery in an eye affected by CMVR. It may produce vitritis, cystoid macular edema, epiretinal membrane, or optic-disc inflammation and can reduce vision despite an inactive necrotizing lesion [40]. Active infection and immune-mediated inflammation can coexist, so the distinction should be based on serial retinal assessment rather than immune recovery alone [7]. New border advancement or satellite lesions support recurrent CMVR; visually significant inflammation after adequate viral control may warrant anti-inflammatory treatment with appropriate antiviral coverage.

The distinction is best made anatomically and longitudinally. Recurrent CMVR typically produces a new or advancing border of retinal whitening, new satellite lesions, or extension from the edge of an old scar. IRU more often produces vitreous inflammation, macular edema, or epiretinal change without an active necrotizing border. OCT, color photography, and, when uncertainty persists, aqueous PCR can help. Corticosteroids should not be escalated solely because vitreous inflammation is present; active CMV should first be reasonably excluded and antiviral coverage ensured.

### 9.2. Retinal Breaks and Detachment

Rhegmatogenous retinal detachment develops because healed CMVR leaves thin, atrophic retina that is vulnerable to breaks during vitreous remodeling. In the five-year ART-era cohort, retinal detachment occurred at 2.3 per 100 eye-years overall and 4.9 per 100 eye-years among patients with newly diagnosed CMVR [3]. More recent multicenter data continue to associate retinal detachment with poor ocular outcomes [41]. Retinal surveillance should therefore continue after retinitis becomes inactive, particularly when lesions are extensive or the patient develops new flashes, floaters, or a visual-field defect.

Retinal breaks may occur within thin necrotic retina or at its junction with surviving tissue, and traction during vitreous remodeling can lead to rhegmatogenous detachment. New flashes, floaters, a curtain-like defect, or an unexplained acuity decline require prompt dilated peripheral examination, with ultrasonography when the fundus is obscured. Established detachment requires vitreoretinal surgical assessment alongside control of active infection; the location and extent of retinitis, macular status, and overall prognosis influence the operative plan [38,41,44,67].

Prophylactic laser demarcation remains a selective rather than routine strategy. In a 2026 cohort from a resource-limited setting, argon laser was applied to 45 eyes in 38 patients with inactive lesions involving at least 25% of the retina; during 3 years of follow-up, no treated eye developed retinal detachment or loss of central visual acuity. By contrast, among 14 eyes presenting with retinal detachment that underwent repair, 6 (42%) experienced additional visual loss and 4 (28.6%) had recurrent detachment [113]. The repaired-detachment group began with a different structural state and is not a valid causal control for prophylactic efficacy. The small selected cohort does not establish a universal indication for demarcation laser. Its potential role requires individualized vitreoretinal assessment, especially where access to emergency repair is limited.

### 9.3. Macular, Vascular, and Optic-Nerve Injury

Macular edema may reflect IRU, continuing infection, vascular leakage, or another comorbidity; OCT and the contemporaneous retinal examination help distinguish these mechanisms. Epiretinal membrane can cause traction and metamorphopsia, whereas post-necrotic macular atrophy represents permanent tissue loss. An OCT angiography study found microvascular and outer-retinal abnormalities beyond clinically visible lesions, supporting structural assessment even when the macula appears uninvolved on color imaging [45]. Additional antiviral exposure cannot restore atrophic retina; progressive edema or traction should prompt cause-specific ophthalmic management [40,44].

Occlusive vasculopathy can produce retinal ischemia, neovascular complications, and vitreous hemorrhage despite suppression of active retinitis [29,79,80]. Fluorescein angiography helps define nonperfusion, leakage, and neovascularization when these findings would alter management. Optic-nerve dysfunction may accompany peripapillary necrosis, ischemia, direct viral involvement, or immune recovery inflammation; direct CMV involvement has been documented histologically in a child with severe combined immunodeficiency [114]. Pupillary responses, color vision, fields, optic-disc assessment, and selected VEP testing can supplement acuity and retinal imaging. Persistent visual loss with an inactive lesion requires assessment for these complications rather than automatic antiviral escalation [44,98].

### 9.4. Maintenance Withdrawal and Continued Surveillance

For HIV-associated CMVR, current NIH guidance requires at least three months of anti-CMV treatment, inactive retinal lesions, and a CD4 count of at least 100 cells/µL sustained for at least three months in response to ART before maintenance is stopped [7]. The decision is made with the treating ophthalmologist. Observational follow-up supports withdrawal after sustained immune recovery, although the eligibility criteria used in individual studies differ [115]. Blood CMV DNA is not an adequate stopping rule.

After maintenance withdrawal, ophthalmic monitoring should occur at least every three months initially and may become annual after sustained immune reconstitution and clinical stability [7]. New symptoms require urgent review between visits. Maintenance should be restarted if the CD4 count falls below 100 cells/µL; a recurrent active retinal lesion requires assessment for reinduction rather than resumption of maintenance alone. This distinction connects the stopping decision back to Figure 1 and Section 8.

No comparable universal immune threshold exists for non-HIV CMVR. Maintenance duration should be linked to the expected trajectory of immunosuppression, systemic CMV control, retinal severity, recurrence history, and the reversibility of the underlying immune defect. Patients continuing intensive chemotherapy, high-dose corticosteroids, JAK inhibition, or post-transplant immunosuppression may require prolonged suppression or repeat induction. The decision should be revisited prospectively at each major change in systemic therapy rather than made once when retinal quiescence is first achieved. Functional immune tests can inform specialist assessment, but neither vaccine responses nor CMV-specific T-cell assays currently provide a validated ocular stopping threshold [37,89,90].

## 10. An Integrated Clinical Decision Framework

### 10.1. Initial Assessment

Use Table 1 to establish the host context and Table 2 to characterize the retinal lesion. Assess immediate vision threat and diagnostic uncertainty while obtaining baseline CBC, renal function, medication history, and relevant immune data. A typical active lesion within immediate sight-threatening distance favors systemic induction plus adjunctive intravitreal therapy; a peripheral active lesion may be managed with systemic therapy and close retinal review. Diagnostic sampling can occur in parallel if uncertainty could change management. Table 3 supports the route and agent choice after these assessments, as shown in Figure 1 [7].

### 10.2. Review During Treatment

At each treatment review, document lesion activity, bilateral findings, vision, actual antiviral exposure, and toxicity. Advancing retinitis prompts the exposure and resistance review in Section 8. If the lesion is inactive but vision worsens, assess IRU, edema, traction, ischemia, detachment, and optic-nerve injury using Section 9 and selected functional tests from Section 6.4. Ophthalmology should communicate the retinal endpoint, while the systemic team specifies CMV kinetics, organ tolerance, and the next planned change in immunosuppression. Table 4 supplies supporting evidence rather than an additional decision pathway.

### 10.3. Maintenance and Follow-Up

When induction has controlled active retinitis, specify the maintenance regimen, next retinal examination, and criteria for urgent reassessment. In HIV, coordinate ART initiation or optimization and use the immune-recovery criteria in Section 9.4. In non-HIV hosts, reconsider duration before renewed chemotherapy, transplantation-related treatment, or corticosteroid escalation. The care plan should identify which team monitors antiviral toxicity and which clinician examines the retina. Withdrawal of maintenance does not end ophthalmic follow-up [7,39,107].

## 11. Special Considerations in Resource-Limited Settings

Implementation evidence from resource-limited settings comes mainly from observational screening and treatment programs. The Myanmar screening study demonstrated that point-of-care dilated examination greatly increased detection among people entering HIV care [14]. The Malawi feasibility study found CMVR despite unreliable visual symptoms and acuity as screening indicators [13], and a Myanmar service cohort documented oral valganciclovir and intravitreal ganciclovir delivery by trained primary HIV clinicians [83]. These studies support integration of retinal assessment into advanced HIV services; they do not establish that every program can reproduce the same outcomes without training, supervision, and referral capacity.

The following are proposed implementation adaptations based on those service data and established treatment principles: define a referral route from HIV and malignancy services to retinal examination, train and supervise clinicians performing fundus assessment or injections, standardize intravitreal preparation, and provide explicit return instructions for pain, reduced vision, and field loss [7,13,14,64,65,83]. Where systemic antiviral access is limited, local treatment may be necessary but leaves fellow-eye and extraocular risk incompletely addressed. Programs should pursue systemic treatment access and immune restoration while recording examination delays, missed visits, and complications. Portable imaging can support consultation when available but should not substitute for an examination that is required urgently.

For a patient newly diagnosed with HIV and active CMVR, anti-CMV therapy should begin promptly and ART should generally start within one to two weeks, with coordination for IRU risk and other opportunistic infections [7]. The recent multidisciplinary case series illustrates this need for coordinated care but does not validate a screening algorithm [116].

## 12. Knowledge Gaps and Research Priorities

Prospective screening research should estimate absolute risks of asymptomatic and sight-threatening CMVR within defined HIV, HSCT, SOT, pediatric, and targeted-therapy populations. Reports should specify ART exposure, immune phenotype, systemic CMV kinetics, screening frequency, and referral pathways. Geographic comparisons require comparable denominators and calendar periods; otherwise differences in case detection may reflect access and ascertainment rather than biologic risk [14,15,16,35,85]. The geographic evidence in Figure 2 therefore identifies data gaps as well as settings with substantial reported burden.

Newer antivirals require ocular pharmacokinetic and prospective treatment studies. Letermovir prophylaxis trials and the systemic maribavir trial answer questions that differ from active retinal infection [60,61]. The six-patient maribavir series included only four eyes with active disease at treatment transition; the remaining seven eyes were already inactive [62]. Future studies should separate induction success from maintenance of quiescence, record concurrent local therapy, and define resistance, toxicity, and visual outcomes prospectively.

Intravitreal dosing studies should distinguish single-injection dose from cumulative weekly exposure and should specify frequency, systemic co-therapy, host immunity, and lesion location. The meta-analysis by Jeong and colleagues provides an association between greater first-week exposure and anatomic resolution, while the broader review by Putera and colleagues demonstrates treatment heterogeneity [54,99]. Neither supports an unqualified claim that a larger injection dose improves vision. Prospective comparisons should report retinal inactivity, visual fields and acuity, recurrence, detachment, and ocular toxicity.

A further priority is to identify more reliable biomarkers of immune readiness for discontinuing maintenance therapy in non-HIV hosts. Total lymphocyte count or CD4 count may incompletely represent CMV-specific immunity after transplantation or targeted therapy. CMV-specific T-cell assays may eventually help guide secondary prophylaxis if they can be technically standardized and validated against ocular relapse. Until then, maintenance duration should remain individualized and conservative when substantial immunosuppression persists. Pediatric and IEI cohorts are particularly needed because cell counts and age-dependent immune maturation complicate extrapolation from adults [32,33,34,35,36,37,89,90].

Finally, visual outcomes should be separated into preventable components. Loss from active foveal necrosis, delayed retinal detachment, macular ischemia, and IRU-associated edema represent different failure modes and require different interventions. A future CMVR registry should record time from symptom onset to examination, lesion zone and extent, treatment-initiation time, serial image-defined activity, immune trajectory, antiviral exposure, resistance mutations, detachment, IRU, and patient-reported visual function. Such data would allow prevention quality to be measured directly rather than inferred.

## 13. Conclusions

CMVR remains a treatable cause of visual loss across advanced HIV, transplantation, treatment for malignancy, targeted immunosuppression, and pediatric immune deficiency. Clinical decisions require the host immune trajectory, retinal activity and location, diagnostic certainty, and treatment tolerance to be assessed together. Geographic studies demonstrate persistent detection gaps, but heterogeneous populations and methods preclude a global prevalence ranking.

Dilated retinal examination and serial imaging anchor diagnosis and monitoring. Ocular PCR resolves selected uncertainties, with negative and positive results interpreted in clinical context. Systemic valganciclovir or ganciclovir remains the usual treatment backbone when feasible, and adjunctive intravitreal therapy provides rapid local exposure for immediately sight-threatening disease. Resistance, intolerance, and limited evidence for newer agents require individualized specialist decisions.

Long-term care should connect induction, maintenance, immune recovery, and continued surveillance. Retinal inactivity does not exclude visual dysfunction from detachment, macular atrophy, ischemia, epiretinal membrane, or optic-nerve injury. Preserving vision therefore requires early diagnosis, safe antiviral exposure, appropriate immune restoration, and timely detection of structural and functional complications.

## Data Availability

No new data were generated in this narrative review. All data discussed are derived from the cited published literature.

## Supplementary Materials

The following supporting information can be downloaded at: https://www.mdpi.com/article/10.3390/jcm15197482/s1. Supplementary File S1: Targeted Literature Search and Source-Verification Strategy.
