# Supplementary material for: Cytomegalovirus Retinitis Across the Immunosuppression Spectrum: A Contemporary Framework for Risk Stratification, Diagnosis, and Vision-Preserving Therapy
Source: J Clin Med. 2026 Sep 25;15(19):7482. doi: 10.3390/jcm15197482 (PMC13665908; doi:10.3390/jcm15197482)
Supplement: Supplementary file 1 [file jcm-15-07482-s001.zip › jcm-4535163-supplementary.pdf]

Supplementary File S1

Targeted Literature Search and Source-Verification Strategy

Cytomegalovirus Retinitis Across the Immunosuppression Spectrum: A Contemporary Framework for Risk Stratification, Diagnosis, and Vision-Preserving Therapy

Manuscript ID: jcm-4535163 | Last search: 20 September 2026

S1.1. Scope and sources

A targeted literature update was performed for this narrative review on 19 September 2026. PubMed, including MEDLINE-indexed and other PubMed records, was searched through the NCBI E-utilities interface. The topic searches used a publication-date boundary until September 2026. PubMed Central and publisher websites were used to retrieve accessible full text. The NIH adult and adolescent opportunistic-infection guideline and relevant consensus statements were checked directly.

The searches supported a focused revision rather than a systematic review. For each topic query, up to 40 records ranked by relevance were retrieved for title/abstract inspection. The ten topic queries returned 351 records before cross-query deduplication and 323 unique PubMed identifiers. These counts describe retrieval for the targeted update; they are not counts from exhaustive screening. Additional reference-list, author/title, DOI, and known-identifier searches were used to locate or verify relevant studies. No claim is made of duplicate independent study selection, formal risk-of-bias scoring, protocol registration, or a new pooled analysis.

S1.2. Selection and use of evidence

English-language human clinical studies relevant to acquired CMVR risk, screening, diagnosis, immune assessment, antiviral management, or ocular outcomes were considered. Randomized trials and prospective cohorts were prioritized for comparative treatment effects and natural history. Retrospective series and case reports were retained where pediatric, inherited immune-deficiency, and salvage-treatment evidence was sparse. Guidelines, consensus statements, and systematic reviews supplied context; numerical study findings were attributed to the corresponding primary report whenever available.

Reports confined to anterior-segment CMV disease, congenital infection without relevant active retinitis, and nonclinical drug development were excluded from the clinical synthesis. General immune and electrophysiology standards were included to define the requested tests and their limitations. They were not treated as direct validation of CMVR treatment thresholds. English-language selection was applied during relevance assessment, rather than through a language filter in the queries. Selection and publication bias remain possible.

S1.3. Executed topic searches

All queries below were executed on 19 September 2026. The database hit count is the number reported by PubMed at execution; records retrieved is the relevance-ranked subset inspected. Search syntax is reproduced as executed. Hit counts can change with subsequent indexing.

Topic	Database hits	Records retrieved
General CMVR literature	2898	40
Ocular PCR and diagnostic uncertainty	247	40
Pediatrics and inborn errors of immunity	124	40
Visual fields and electrophysiology in CMVR	55	40
Geographic clinical evidence	62	40
General immune-function assessment	80	40
CMV-specific cellular immunity	18	18
Clinical electrophysiology standards	124	40
Structural and vascular complications	346	40
Rare immune-deficiency presentations	13	13

S1.3.1. General CMVR literature

"Cytomegalovirus Retinitis"[Mesh] OR "cytomegalovirus retinitis"[tiab] OR "CMV retinitis"[tiab] AND ("1900/01/01"[Date - Publication] : "2026/09/19"[Date - Publication])

PubMed hits: 2898. Retrieved: 40.

S1.3.2. Ocular PCR and diagnostic uncertainty

("cytomegalovirus retinitis"[tiab] OR "infectious uveitis"[tiab]) AND (PCR[tiab] OR "polymerase chain reaction"[tiab]) AND ("1900/01/01"[Date - Publication] : "2026/09/19"[Date - Publication])

PubMed hits: 247. Retrieved: 40.

S1.3.3. Pediatrics and inborn errors of immunity

("cytomegalovirus retinitis"[tiab] OR "CMV retinitis"[tiab]) AND (child*[tiab] OR pediatric*[tiab] OR paediatric*[tiab] OR "primary immunodeficien*"[tiab] OR "inborn errors"[tiab] OR "combined immunodeficiency"[tiab]) AND ("1900/01/01"[Date - Publication] : "2026/09/19"[Date - Publication])

PubMed hits: 124. Retrieved: 40.

S1.3.4. Visual fields and electrophysiology in CMVR

("cytomegalovirus retinitis"[tiab] OR "CMV retinitis"[tiab]) AND (electroretinogra*[tiab] OR "visual field"[tiab] OR "evoked potential"[tiab] OR "multifocal ERG"[tiab]) AND ("1900/01/01"[Date - Publication] : "2026/09/19"[Date - Publication])

PubMed hits: 55. Retrieved: 40.

S1.3.5. Geographic clinical evidence

("cytomegalovirus retinitis"[tiab] OR "CMV retinitis"[tiab]) AND (Myanmar[tiab] OR Thailand[tiab] OR Malawi[tiab] OR Uganda[tiab] OR Brazil[tiab] OR India[tiab]) AND ("1900/01/01"[Date - Publication] : "2026/09/19"[Date - Publication])

PubMed hits: 62. Retrieved: 40.

S1.3.6. General immune-function assessment

("primary immunodeficiency"[tiab] OR "inborn errors of immunity"[tiab]) AND ("laboratory evaluation"[tiab] OR "diagnostic tests"[tiab] OR "vaccine responses"[tiab]) AND ("1900/01/01"[Date - Publication] : "2026/09/19"[Date - Publication])

PubMed hits: 80. Retrieved: 40.

S1.3.7. CMV-specific cellular immunity

"cytomegalovirus"[tiab] AND "cell-mediated immunity"[tiab] AND (guideline*[tiab] OR consensus[tiab] OR randomized[tiab]) AND ("1900/01/01"[Date - Publication] : "2026/09/19"[Date - Publication])

PubMed hits: 18. Retrieved: 18.

S1.3.8. Clinical electrophysiology standards

ISCEV[tiab] AND ("full-field"[tiab] OR "multifocal"[tiab] OR "visual evoked"[tiab]) AND standard[tiab] AND ("1900/01/01"[Date - Publication] : "2026/09/19"[Date - Publication])

PubMed hits: 124. Retrieved: 40.

S1.3.9. Structural and vascular complications

("cytomegalovirus retinitis"[tiab] OR "CMV retinitis"[tiab]) AND ("retinal detachment"[tiab] OR "macular edema"[tiab] OR "epiretinal membrane"[tiab] OR "optic nerve"[tiab]) AND ("1900/01/01"[Date - Publication] : "2026/09/19"[Date - Publication])

PubMed hits: 346. Retrieved: 40.

S1.3.10. Rare immune-deficiency presentations

("cytomegalovirus retinitis"[tiab] OR "CMV retinitis"[tiab]) AND ("combined immunodeficiency"[tiab] OR "inborn errors"[tiab] OR "primary immunodeficiency"[tiab] OR "CD40"[tiab]) AND ("1900/01/01"[Date - Publication] : "2026/09/19"[Date - Publication])

PubMed hits: 13. Retrieved: 13.

S1.4. Supplemental identification and verification

The following additional queries were executed during the same update. Up to 12 records were retrieved for each query, and records unrelated to the intended clinical topic were not used. A zero-result exact-title query was

followed by known-identifier retrieval where an article had already been identified. In particular, the primary immunodeficiency practice parameter was retrieved as PMID 26371839. The broad exploratory PCR query containing “23%” did not serve as evidence for an activity threshold; the relevant ocular-PCR source was subsequently checked directly.

Harper[Author] AND polymerase[Title]

PubMed hits: 19. Records retrieved: 12.

"Practice parameter for the diagnosis and management of primary immunodeficiency"[Title]

PubMed hits: 0. Records retrieved: 0.

ISCEV[Title] AND multifocal[Title] AND 2021[Title]

PubMed hits: 1. Records retrieved: 1.

SANRA[Title]

PubMed hits: 4. Records retrieved: 4.

"Routine immediate eye examination"[Title] OR ("cytomegalovirus retinitis"[Title] AND Myanmar[Title])

PubMed hits: 3. Records retrieved: 3.

"cytomegalovirus retinitis" AND "23%"

PubMed hits: 133. Records retrieved: 12.

"10.12701/jyms.2026.43.13"[AID]

PubMed hits: 1. Records retrieved: 1.

"10.1016/j.ajoint.2026.100247"[AID]

PubMed hits: 1. Records retrieved: 1.

Direct known-identifier retrieval also checked PMIDs 11920309, 11262214, 19632953, 21742304, and 26371839. The original 89 reference entries were matched to PubMed bibliographic records using title/DOI information. Publisher metadata was used where the issue year or article identifier needed clarification. For example, the Microorganisms review by Munro et al. is cited as 2020, volume 8, article 55, following its publisher citation despite online release in December 2019.

S1.5. Numerical and figure source checks

Content	Source checked	Interpretive constraint
Five-year CMVR outcomes	Jabs et al. 2010; PMID 20673591	Event rates retain their person-year or eye-year denominators.
Valganciclovir comparison	Martin et al. 2002; PMID 11948271	Oral valganciclovir vs. intravenous ganciclovir induction; no randomized placebo maintenance arm.
Intravitreal dose synthesis	Jeong et al. 2026; PMID 41530976	First-week exposure association from heterogeneous studies; no causal visual-benefit claim.
Maribavir	SOLSTICE trial, 2025 transplant consensus, 2026 CMVR series	The systemic trial excluded retinitis; four active eyes in the series are separated from seven already inactive eyes.
Geographic Figure 2	Seven primary studies cited in the revised figure and legend	Selected populations and different endpoints; no global prevalence estimate or hotspot ranking.

Figure 1 is an author-constructed clinical synthesis linked to the cited guidance. Figure 2 reproduces source-specific study findings without new pooling, simulated curves, cross-study risk ratios, or national extrapolation. The base map uses public-domain Natural Earth boundaries. The full reference details are in the main manuscript; numbering follows the revised manuscript.

S1.6. Reporting reference

Baethge C, Goldbeck-Wood S, Mertens S. SANRA: a scale for the quality assessment of narrative review articles. Research Integrity and Peer Review. 2019;4:5. https://doi.org/10.1186/s41073-019-0064-8
